# Prediction of Facial Emotion Recognition Ability in Patients With First-Episode Schizophrenia Using Amplitude of Low-Frequency Fluctuation-Based Support Vector Regression Model

**DOI:** 10.3389/fpsyt.2022.905246

**Published:** 2022-07-13

**Authors:** Qi-Jie Kuang, Su-Miao Zhou, Yi Liu, Hua-Wang Wu, Tai-Yong Bi, Sheng-Lin She, Ying-Jun Zheng

**Affiliations:** ^1^Department of Psychiatry, The Affiliated Brain Hospital of Guangzhou Medical University, Guangzhou, China; ^2^Centre for Mental Health Research in School of Management, Zunyi Medical University, Zunyi, China

**Keywords:** first-episode schizophrenia (FSZ), facial emotion recognition (FER), functional magnetic resonance imaging (fMRI), amplitude of low-frequency fluctuation (ALFF), support vector regression (SVR)

## Abstract

**Objective:**

There were few studies that had attempted to predict facial emotion recognition (FER) ability at the individual level in schizophrenia patients. In this study, we developed a model for the prediction of FER ability in Chinese Han patients with the first-episode schizophrenia (FSZ).

**Materials and Methods:**

A total of 28 patients with FSZ and 33 healthy controls (HCs) were recruited. All subjects underwent resting-state fMRI (rs-fMRI). The amplitude of low-frequency fluctuation (ALFF) method was selected to analyze voxel-level spontaneous neuronal activity. The visual search experiments were selected to evaluate the FER, while the support vector regression (SVR) model was selected to develop a model based on individual rs-fMRI brain scan.

**Results:**

Group difference in FER ability showed statistical significance (*P* < 0.05). In FSZ patients, increased mALFF value were observed in the limbic lobe and frontal lobe, while decreased mALFF value were observed in the frontal lobe, parietal lobe, and occipital lobe (*P* < 0.05, AlphaSim correction). SVR analysis showed that abnormal spontaneous activity in multiple brain regions, especially in the right posterior cingulate, right precuneus, and left calcarine could effectively predict fearful FER accuracy (*r* = 0.64, *P* = 0.011) in patients.

**Conclusion:**

Our study provides an evidence that abnormal spontaneous activity in specific brain regions may serve as a predictive biomarker for fearful FER ability in schizophrenia.

## Introduction

Schizophrenia is a psychiatric disorder characterized by positive symptoms, negative symptoms, and cognitive impairments. Schizophrenia affects nearly 1% of the human population and is ranked as the top 10 causes of disability worldwide (1). Nearly, two-thirds of patients with schizophrenia have difficulty in fulfilling their basic social roles and thus result in a huge burden on families and society. The economic burden of schizophrenia was estimated to range from 0.02 to 1.65% of the gross domestic product. In conclusion, this has a serious impact on the economic burden on countries around the world (2).

Cognitive impairments in schizophrenia include neurocognitive impairment and social cognitive impairment. Neurocognitive impairment involves impaired attention, executive function, working memory, abstract thinking, and the integration of information. Social cognition is defined as the ability to perceive other people’s intentions and temperament, and subsequently guides social activities, thus is of core importance in the understanding of other people’s expressions, characteristics, relationships, and behavior (3).

Social cognition requires higher mental processing, including emotion recognition, theory of mind, attribution bias, and social perception (4). Facial expressions could convey the emotional state of the self, help person to judge the mental state of others, and also affect the generation and regulation of emotional states (5). Therefore, facial emotion recognition (FER) plays an important role in social interactions. Previous studies have reported impaired FER in both patients with first-episode schizophrenia (FSZ) and chronic schizophrenia (6, 7). Additionally, Bulgari et al. found that facial emotion recognition in schizophrenia patients with violent behavior is different from that of non-violent patients, suggesting that face emotion recognition may be associated with violent behavior (8). These findings provide an evidence that FER deficiency in schizophrenia may suggest an inextricably linked social communication impairment.

Neuroimaging is a promising translational tool that has been widely used to characterize brain features. Resting-state functional magnetic resonance imaging (rs-fMRI) is a non-invasive tool with easily obtainable convenience to assess regional and neural function activity at rest. Rs-fMRI methods include amplitude of low-frequency fluctuation (ALFF) (9), regional homogeneity (ReHo) (10), and functional connectivity (FC). Amplitude of low-frequency fluctuation methods could directly reflect the level of spontaneous neuronal activity in each voxel from an energetic perspective (9). Previous studies showed alterations of ALFF values in several brain regions in patients with schizophrenia compared with healthy controls (11, 12), suggesting that ALFF values may be a schizophrenia-associated biomarker. In addition, studies showed that altered ALFF values in various brain regions associated with neurocognitive impairment and social cognitive impairment in schizophrenia patients (13, 14). This suggests an association between cognitive impairment and neuropathological changes of brain region.

Support vector machine (SVM) methods have been increasingly applied in the classification of psychiatric disorders combining neuroimaging data. Support vector regression (SVR) is an extension of SVM, a well-established predictive model applied in neuropsychiatric disorders. SVR permits the quantitative prediction of a variable of interest without the need for a discrete categorical decision, allowing exploration of outcome on a gradual scale (15). The parameters in the SVR model represent the weighted contribution of all features.

Previous studies have shown that MRI-based SVR models could effectively predict brain disorders associated features. For instance, previous studies found elevated brain age and accelerated aging in patients with schizophrenia using SVR method (16, 17). Ouyang et al. (18) found that neonatal cortical microstructure exhibited high selectivity and robustness in prediction of individual cognition and language function at the age of 2 years by using the diffusion-MRI-based SVR model. The combination of clinical characteristics and structural MRI data before treatment in SVR model could improve the predictive accuracy for ECT remission in patients with schizophrenia (19). Gong et al. showed that multi-parametric MRI-based SVR model using both structure and diffusion MRI data may predict individual response to ECT therapy and showed a higher predictive accuracy than single-modal MRI-based SVR model (20).

Predictive studies of MRI data for clinical features have attracted our attention, which showed that MRI data from patients with bipolar disorder in depressive episodes could predict the number of depressive episodes and that gray matter volume in patients with Alzheimer’s disease and ALFF values in patients with Parkinson’s disease predicted their clinical symptom scores (21–23). In addition, de Wit et al. (24) predicted functioning, negative, and disorganization symptom levels at 6 years from baseline levels of MRI data in adolescents at ultrahigh risk for psychosis. These results have shown that SVR models based on MRI data (e.g., ALFF, GMV, etc.) can effectively predict quantitative clinical indicators at the individual level.

However, a few studies had attempted to predict FER ability at the individual level in schizophrenia patients. Our aim in this study was 2-fold: first, we tested whether the application of SVR techniques to individual-level rs-fMRI could accurately predict FER ability in FSZ patients; and second, we explored which brain regions were closely associated with the current prediction. We hypothesized that individual-level ALFF values could effectively predict FER ability in FSZ patients and that the brain regions contributed to such prediction were several regions known to be associated with FER.

## Materials and Methods

### Subjects

The inclusion criteria for the FSZ patients were as follows: (1) meeting the diagnostic criteria for schizophrenia in the *Diagnostic and Statistical Manual of Mental Disorders, Fourth Edition* (DSM-IV); (2) first episode and duration of psychosis less than 2 years; (3) aged 18–50 years; (4) psychiatric medication treatment for no more than 6 months; (5) total scores on the Positive and Negative Syndrome Scale (PANSS) over 50 points (25); (6) vision or corrected vision to 1.0 or above; (7) formal education no less than 6 years; (8) no history of electroconvulsive therapy (ECT) within 2 years; (9) no history of alcohol or drug abuse within the past 5 years; and (10) written informed consent from the patient or their legal guardian. Exclusion criteria: (1) meeting the diagnostic criteria for other psychiatric disorders in the DSM-IV; (2) history of organic brain disease such as epilepsy, traumatic brain injury, severe encephalitis, and brain tumor; and (3) contraindications for MRI. Inclusion criteria for HC group were as follows: (1) no diagnostic history or family history of psychiatric disorders; (2) aged 18–50 years; (3) vision or corrected vision to 1.0 or above; (4) formal education no less than 6 years; (5) psychiatric medication free; and (6) written informed consent obtained from the subjects. The exclusion criteria were the same as those for the patient group. According to the inclusion and exclusion criteria, 31 patients with FSZ and 33 with HCs were included. However, three FSZ patients were excluded in the further analysis process due to failing to complete the FER-related visual search task. This study was approved by the Institutional Review Board of the Affiliated Brain Hospital of Guangzhou Medical University. In addition, written informed consent was obtained from all subjects in accordance with the Declaration of Helsinki.

### Procedures

#### Psychophysical Experiment

**FIGURE 1:**
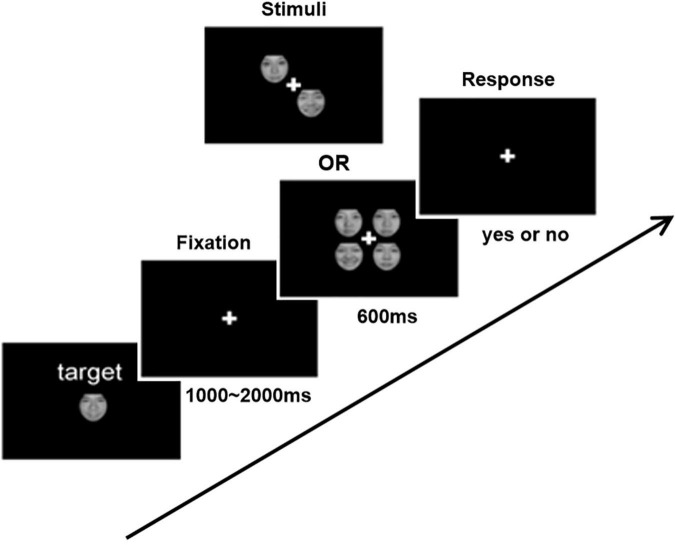
Schematic demonstration of the experimental procedure for emotional face search task. In each trial, two or four items with or without the emotional face were presented. Subjects were asked to respond whether the target was presented or not.

The experimental procedure refers to a previous study (26). Each subject was required to complete two visual search experiments (happy FER and fearful FER) to obtain individual FER ability. The separate procedure of each experiment was shown in Figure 1. Each experiment contains three phases. The first phase was a waiting period for the presentation of a small white cross in the center of the monitor for 1,000–2,000 ms. The second phase was a stimulation period for the presentation of two or four faces in the center of the monitor for 600 ms. Faces with emotional expression appeared randomly in half of the trials. All faces used in the experiments were selected from the Chinese Facial Affective Picture System (CFAPS) (27), including 32 happy faces, 32 fearful faces, and 64 neutral faces. All faces were pre-processed in Photoshop to exclude confounding factors such as the hair, ear, and facial contour before experiments. The third phase was a reaction period. The subjects were required to judge as quickly and accurately as possible whether the presented faces contained emotional expression immediately after the faces disappeared (emotional face press “n”; non-emotional face press “m”). Above is a whole cycle in the experimental procedure. Each experiment contains 6 blocks, and each block contains 40 cycles. All subjects were asked to complete the happy FER experiment first and then the fearful FER experiment. To obtain an adequate relax, sufficient rest time was ensured between the two experiments. Notably, all subjects were given appropriate instructions and enough practice to understand the experiment before the experiment.

#### Resting-State Functional Magnetic Resonance Imaging Acquisition and Processing

The MRI data collection was performed on the same day as psychophysical experiment. The collection of the imaging data was performed at the Department of Radiology of the Affiliated Brain Hospital of Guangzhou Medical University by an attending level or higher professional technician. All subjects were scanned using a Philips 3.0T MRI system, and the signals were received by an eight-channel SENSE head coil. During the scan, the subjects were asked to secure their heads with supporting foam pads and to minimize the noise with rubber ear buds. Whole-brain rs-fMRI scans comprising a total of 240 echo-planar imaging sequences were obtained with the following parameters: TR = 2,000 ms, TE = 30 ms, flip angle = 90°, matrix = 64 × 64, slice thickness = 4 mm, slice number = 33, voxel size = 3.44 mm × 3.44 mm × 4.60 mm, slice gap = 4.6 mm, and field of view (FOV) = 192 mm × 192 mm.

The rs-fMRI data were analyzed and processed using RESTplus (28) in MATLAB 2013b (MathWorks Inc.). The ALFF method was selected to calculate voxel-level spontaneous neuronal activity in further analysis. The fMRI data require head movements of less than 2 mm in translation and less than 2° in rotation. The pre-processing included removing the first 10 dummy scans, slice timing correction, realignment, spatial standardization (normalized by using the conventional EPI template), smoothened with an 8-mm full width at half-maximum Gaussian kernel, detrending, and nuisance covariate regression. To calculate the ALFF, a fast Fourier transform (FFT) was calculated first. The FFT converted the time series of the whole-brain signal into a frequency domain power spectrum. Then, the square root of the power spectrum at each frequency was averaged across the filtered band (0.01–0.08 Hz). The ALFF of the whole-brain signal was obtained. The ALFF of each voxel was then divided by the global mean of the ALFF (mALFF) to eliminate the differences in the whole-brain ALFF at the overall level among the individuals.

#### Facial Emotion Recognition Ability Prediction

The prediction of FER ability of schizophrenia was performed in MATLAB (MathWorks Inc.) using the SVR method with the custom MATLAB scripts. In this study, the SVR analysis was based on the voxel-level mALFF value. The relevance method was used for feature extraction.

The brain regions that showed difference in the group comparison were selected as the mask for the SVR analysis, and a total of 1,664 voxels were selected. The feature algorithms were conducted in MATLAB 2013b. All features with 1,664 voxels for each patient (28 × 1,664) were concatenated into a feature vector and connected in parallel to form a mALFF feature matrix. The relevance method was used for feature selection with a threshold value less than 0.05. The relevance method is a method of improving prediction accuracy by first screening out voxels that are more relevant to the predictor variables through Pearson correlation analysis. Therefore, the Shapiro–Wilk test was used to evaluate whether each continuous variable following the normal distribution before SVR analyses for using the Pearson correlation coefficient. In this test, the SVR predictions were performed based on MRI data from patients with FSZ only with feature selection.

For the purpose to select the best model for the prediction of individual FER ability, we conducted leave-one-out cross-validation (LOOCV). In LOOCV, one patient was used as a testing sample, and the remaining patients were used as training samples to select the features and build the model. This processing was repeated until all patients became a testing sample. This process was repeated 28 times in this study to obtain 28 predicted scores. Two standard statistical variables were selected as the SVR indices, including the mean squared error (MSE) and Pearson’s correlation coefficient (*r*). Both MSE and Pearson’s correlation coefficient were calculated based on the difference between the predicted value and the actual value. The Pearson correlation coefficient was the SVR accuracy. To confirm the effectiveness of the prediction for FER ability by using the mALFF value, a permutation test was applied. We fixed the FER ability and permuted all mALFF values randomly. This process was repeated 1,000 times to generate 1,000 datasets with random mALFF values. All random mALFF values could create a new MSE and *r*. The *P*-value of *r* in LOOCV prediction was calculated as the ratio of number of new *r* greater than *r* in LOOCV prediction over number of all permutation tests. However, the *P*-value of MSE was calculated as the ratio of number of new MSE lower than MSE in LOOCV prediction over number of all permutation tests. The *P*-value of the permutation tests was required less than 0.05.

### Statistical Analysis

Independent-sample *t*-tests and chi-square tests were used for the group comparison of the basic demographic and descriptive characteristics. The psychophysical experiment is a 2 (stimulus: happy/fearful faces) × 2 (subject group: FSZ/HC) mixed design, where the dependent variable was accuracy. The stimulation was a within-subject factor and the group (FSZ or HC) was a between-subject factor. Given two independent visual search experiments in our study, we used a Bonferroni correction to minimize the risk of type I errors, and thus the two-tailed significance threshold in multiple testing was set at 0.025. The effect size (ES) was provided for each statistical test. In rs-fMRI, we used a general linear model to construct a *t*-test for the difference of mALFF value between the two groups while regressing to remove the effect of sex, age, and education. The AlphaSim method was used for correction with a *P* < 0.05. In SVR, only the model with a *P*-value of the permutation test less than 0.05 was significant, which requires the constructed model to have the *r* greater than 95% new *r* and the MSE less than 95% new MSE. All values are represented by means ± SD, and all statistical tests were conducted by two-tailed tests with 0.05 as the level of significance (α). The statistical analysis was performed using the SPSS version 22.0.

## Results

### Demographic and Clinical Characteristics of the Subjects

The two groups showed no difference in general demographic features including gender (χ^2^ = 0.46, *P* = 0.500), age (*t* = 0.59, *P* = 0.557), or education (*t* = 0.42, *P* = 0.674). The chlorpromazine equivalent doses of patients with FSZ were calculated according to Gardner et al. (29). The general demographics of the participants and the descriptive characteristics of the patients are shown in Table 1.

**TABLE 1:** Information on demographic, clinical characteristics, and facial emotion recognition (FER) accuracy of the participants.

	Patients with FSZ (*n* = 28)	HC (*n* = 33)
Gender: Male in% (#)	57.1 (16)	48.5 (16)
Age (years)	25.1 ± 6.9	24.2 ± 5.0
Education (years)	11.3 ± 3.3	11.7 ± 3.1
Duration of illness (months)	8.7 ± 7.2	NA
Antipsychotics drug use (mg)^1^	217.4 ± 230.4	NA
PANSS positive	17.4 ± 5.2	NA
PANSS negative	13.6 ± 4.3	NA
PANSS psychopathology symptoms	34.1 ± 8.8	NA
PANSS total	65.1 ± 15.5	NA
Happy FER accuracy*	0.89 ± 0.06	0.94 ± 0.06
Fearful FER accuracy**	0.74 ± 0.10	0.86 ± 0.07

*^1^Chlorpromazine equivalent doses were calculated.*

*FSZ, first-episode schizophrenia; HC, healthy control; NA, not applicable; PANSS, positive and negative syndrome scale; FER, facial emotion recognition.*

*Significantly different between the patient and control group after Bonferroni adjustments (P < 0.05/2).

**Significantly different between the patient and control group after Bonferroni adjustments (P < 0.01/2).

### Behavioral Performance of the Visual Search Task

In the psychophysical experiment, only trials with reaction time less than 3 s were included in the further analysis (26). The accuracy of the two stimuli was then calculated as the ratio of the correct responses number to the total number of all trials. We focused on the difference in FER accuracy between the two groups in different facial emotion. The interaction between the stimulus and the subject group was significant, *F* = 6.652, *P* = 0.011, ES = 0.663, indicating that the degree of FER accuracy influenced by the stimulus differed between the two groups. A simple effects analysis between the stimulus and subject group showed a significant stimulus effect after controlling for the subject group factor (patients: *F* = 50.721, *P* < 0.001, ES = 1.830; healthy controls: *F* = 15.405, *P* < 0.001, ES = 1.009), suggesting that subjects had more difficulty identifying fearful faces than happy faces. In addition, the subject group effect was equally significant after controlling for the stimulus factor (happy: *F* = 6.091, *P* = 0.015, ES = 0.634; fearful: *F* = 37.398, *P* < 0.001, ES = 1.571), suggesting that the FER accuracy was impaired in patients with FSZ compared to healthy controls (refer to details in Table 1).

### Comparison of Mean of the Amplitude of Low-Frequency Fluctuation Between the Groups

Through the calculation of the RESTplus software (28), a threshold of *P* < 0.05 after AlphaSim correction required a threshold of *P* < 0.001, uncorrected with cluster size of 60 voxels.

**FIGURE 2:**
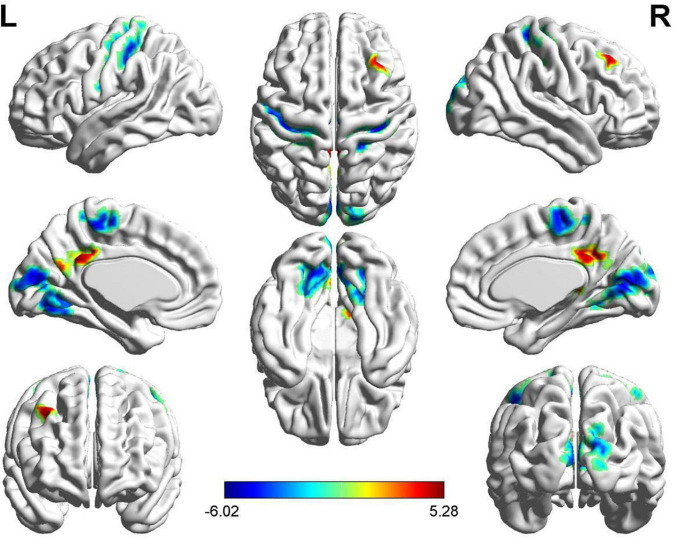
The brain regions with different mean of the ALFF (mALFF) value between patients with first-episode schizophrenia (FSZ) and healthy control (HC). In patients, increased mALFF value were distributed in regions including medial cingulate, posterior cingulate, precuneus, and middle frontal gyrus (*P* < 0.05, AlphaSim correction), while decreased mALFF value in calcarine, lingual gyrus, cuneus, precentral gyrus, postcentral gyrus, paracentral lobule lobes, supplementary motor area, and superior occipital gyrus (*P* < 0.05, AlphaSim correction).

Compared with HC subjects, increased mALFF values were distributed in the limbic lobe and frontal lobe, including the bilateral medial cingulate, right posterior cingulate, right precuneus, and right middle frontal gyrus in FSZ (*P* < 0.05, AlphaSim correction). However, decreased mALFF values were observed in the frontal lobe, parietal lobe, and occipital lobe, including the bilateral calcarine, bilateral lingual gyrus, bilateral cuneus, bilateral precentral gyrus, bilateral postcentral gyrus, left paracentral lobule lobes, right supplementary motor area, and right superior occipital gyrus (*P* < 0.05, AlphaSim correction) (refer to details in Figure 2 and Table 2).

**TABLE 2:** The different brain regions of mean of the ALFF (mALFF) value between two groups.

Brain region (AAL)	Peak MNI coordinates			BA	Cluster size (voxels)
	x	y	z		
**Schizophrenia > HC**					
Bilateral medial cingulate/right posterior cingulate/right precuneus**	9	−45	33	31	220
Right middle frontal gyrus*	36	30	45	8	63
**HC > Schizophrenia**					
Bilateral calcarine/bilateral lingual gyrus/left cuneus**	−3	−84	15	18	533
Left precentral gyrus/left postcentral gyru**	−39	−24	39	3	380
Right precentral gyrus/right postcentral gyru**	36	−21	48	−	209
Left paracentral lobule lobes/right supplementary motor area**	3	−24	63	6	173
Right superior occipital gyrus/right cuneus*	15	−93	6	−	86

*AAL, anatomical automatic labeling; MNI, Montreal Neurological Institute; BA, Brodmann area.*

*Significantly different between the patient and control group (P < 0.05).

**Significantly different between the patient and control group (P < 0.01).

### Support Vector Regression and Correlation Analysis

**FIGURE 3:**
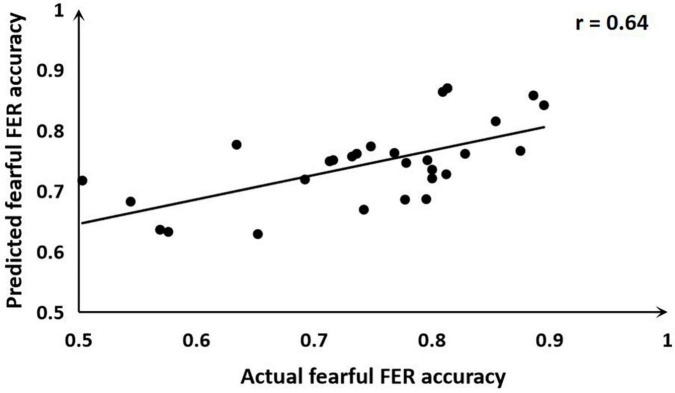
Scatter plot showing the prediction for fearful facial emotion recognition (FER) accuracy in each subject derived from resting-state functional magnetic resonance imaging (rs-fMRI) data using the support vector regression (SVR) analysis, compared with their actual fearful FER accuracy.

The ALFF-based SVR allowed for the quantitative prediction of fearful FER accuracy with statistically significant accuracy of 64% (*r* = 0.64, *P* = 0.011; MSE = 0.01, *P* = 0.011; Figure 3). Predicted and actual accuracy for FER were demonstrated at the individual level. In addition, a weight map is generated at the end of the SVR concerning the survival of the features. The prediction was based on functional alterations across multiple brain regions, especially in the right posterior cingulate/right precuneus (weight = 0.96) and the left calcarine (weight = 0.89). The results are described in detail in Table 3. The correlation analysis showed that the mALFF signals of both the right posterior cingulate/right precuneus (*r* = 0.47, *P* = 0.011) and the left calcarine (*r* = 0.40, *P* = 0.037) were positively correlated with the fearful FER accuracy. However, the mALFF-based SVR model performed poorly in predicting happy FER accuracy in FSZ patients (*r* = 0.01, *P* = 0.990; MSE = 0.01, *P* = 0.882).

**TABLE 3:** The brain regions closely related to the fearful facial emotion recognition (FER) accuracy in patients with first-episode schizophrenia (FSZ).

Brain region (AAL)	Peak MNI coordinates			Weight	Cluster size (voxels)
	x	y	z		
Right posterior cingulate/right precuneus*	9	−48	24	0.96	24
Left calcarine*	−6	−75	6	0.89	19

*Significantly different between the patient and control group (P < 0.05).

## Discussion

To the best of our knowledge, this is the first study using SVR model based on the rs-fMRI mALFF method to predict the individual FER ability in patients with FSZ. Multiple brain regions were included in this SVR model, especially ALFF in the limbic lobe and occipital lobe in patients could effectively predict fearful FER accuracy.

In the behavioral test, similar to the findings of most previous studies, our test showed that patients with FSZ performed worse in FER than HCs (7, 14). Furthermore, only the interaction between stimulus and group was significant, which indicated that the accuracy of FER in schizophrenia was impaired more severely for fearful facial emotion than happy facial emotion. Previous studies showing that in schizophrenia, negative FER (30, 31), including fearful emotion, impaired more severe than positive FER. Bonfils et al. (32) showed that patients with FSZ had the highest accuracy in recognizing happy facial emotion and the least accuracy in recognizing fearful facial emotion, which suggested FSZ patients had more difficulty in identifying fearful facial emotion.

The results of rs-fMRI showed that ALFF values increased in the limbic and prefrontal lobes and decreased in the frontal, parietal, and occipital lobes. Gong et al. (11) found that the ALFF value decreased in brain regions of the post-central gyrus, left inferior parietal gyrus, and right occipital gyrus, while the ALFF value increased in brain regions of the right inferior frontal gyrus, left inferior temporal gyrus, and right cingulate cortex in schizophrenia patients. Our study showed results that are consistent with previous studies (11, 12). This suggests that the ALFF alterations in brain regions were stable in schizophrenia patients and might be used as predictors for clinical features.

In this study, we chose SVR that is the most classical quantitative prediction method, with high prediction accuracy and good generalization ability. Previous similar studies have shown that whole-brain mALFF could be used as input features for predicting clinical scores (21, 33). The SVR results of our study found that spontaneous activities in the specific brain regions, including spontaneous activity in the right posterior cingulate gyrus, right precuneus gyrus, and left calcarine, showed effectiveness in predicting the fearful FER accuracy in schizophrenia patients. The cingulate cortex is involved in emotion recognition/perception and regulation. On the contrary, the precuneus is one of the core brain regions for vision-related activities and plays a crucial role in higher visual cognitive functions such as visuospatial tasks, stimulus integration, and retrieval of situational memories (34). In healthy population, brain activities in the posterior cingulate gyrus and precuneus changed in response to different emotional conditions (35, 36). Furthermore, gray matter volume in brain regions such as the precuneus and superior temporal sulcus contributes to the decoding of facial expressions through the mediation effect of facial recognition abilities (37). Previously, a study has reported an association between anger FER ability and rs-fMRI FC between the amygdala and the posterior cingulate gyrus in non-preterm individuals (38). In studies of psychopaths, task-related

fMRI and structural MRI researches demonstrated association between FER and effective connectivity in the amygdala, precuneus, and inferior parietal lobes (39, 40). A fMRI study showed that previous processing stages in the fusiform and posterior cingulate cortex might contribute to amygdala activity (41). This finding may suggest an indirect role for the posterior cingulate cortex in fearful FER processing. Another task-related fMRI study reported that FSZ patients showed hyperactivation in the posterior cingulate and precuneus during FER processing (42). In addition, previous review showed the importance of the interactions among brain regions such as the posterior cingulate and the ventral medial prefrontal cortex in FER and theory of mind ability and other aspects of social cognition (43). The calcarine is the primary visual cortex and involved in the early stages of visual image processing and the perception of visual cues (44). Researches in healthy populations found that the visual cortex, specially the calcarine, plays a crucial role in fearful conditioning (45). A facial emotion discrimination study in patients with first-episode psychosis showed that brain regions such as the bilateral lingual gyrus and calcarine were involved in facial perception (46). In addition, previous research showed that the implication of calcarine in visual information processing and exploratory eye movement in patients with schizophrenia (47). The main brain regions involved in this SVR model that contributed to the best prediction for fearful FER accuracy are attributable to the facial emotions and vision processing associated regions. In the correlation analysis, spontaneous activity in the right posterior cingulate/right precuneus gyrus and left calcarine gyrus were positively correlated with the fearful FER accuracy in schizophrenia patients, which in turn supporting our SVR analysis result.

Our results emphasized the reliability of the SVR model. It should be noted that in the SVR method, the brain regions with higher predictive effectiveness mentioned above may present high discriminative power due to two possible reasons. First, these brain regions are able to distinguish signal intensity between individuals with low and high scores. In addition, different brain regions with the ability to distinguish between low and high scores can synergize with each other. Therefore, regions identified in this study could be regarded as a brain network model rather than as independent areas (21).

Unfortunately, the SVR model based on spontaneous activity in specific brain regions was a poor predictor of happy FER ability in schizophrenia patients. There are two possible reasons for this. First, the mask used for prediction in this study was based on brain regions that differed in ALFF between the two groups. Therefore, more differences in fearful FER between the two groups might be more associated with ALFF differential brain regions. Second, previous studies have shown that fearful emotions are more correlated with spontaneous neuronal activity of resting brain (48). However, few studies suggest a correlation between happy emotion and ALFF.

### Strengths and Limitations

Our study has some strengths compared to previous similar studies. First, our study included FSZ patients, which reduced the confounding effect of disease duration on the results. Second, in the behavioral test, we adopted a visual search task to behaviorally measure the FER ability in schizophrenia, which is more concerned with the detection and identification of stimuli compared with the match-to-sample task (49). Third, rs-fMRI has the advantages of short scan time, objective interpretation, and reproducibility compared to task fMRI. In addition, unlike previous studies on the FER ability of FSZ patients, this is the first study using SVR model based on the rs-fMRI mALFF method to predict the individual FER ability in patients with FSZ, with some success.

However, limitations of this study should be noted. First, all participants were surveyed using a cross-sectional design, which only explored the predictors of the current FER ability. Follow-up studies are needed to explore the predictors of FER deficiency progression in schizophrenia patients. Second, structural MRI was not performed. Structural MRI scans can measure some changes in the gray and white matter of the brain that have been found to correlate with FER ability (14, 50). Moreover, our study is a single-center project and therefore has a single schizophrenic disease profile. Therefore, further studies in the future using a more representative sample would be required to better characterize the potential of resting-state fMRI as a clinically useful diagnostic aid.

## Conclusion

Our study indicates that abnormal spontaneous activity in multiple brain regions could effectively predict the fearful FER accuracy. This aid has the potential to help clinical evaluation, which is difficult using traditional methods alone. However, prediction accuracy requires a greater accuracy of prediction before it can be put into clinical application. With methods such as improving in prediction strategies and combining different types of neuroimaging data, future studies may have a greater accuracy of prediction. Overall, these findings may provide evidence that spontaneous activity in the specific brain regions could be considered a biomarker for fearful FER ability prediction in schizophrenia.

## Data Availability Statement

The original contributions presented in this study are included in the article/supplementary material, further inquiries can be directed to the corresponding authors.

## Ethics Statement

The studies involving human participants were reviewed and approved by the Institutional Review Board of the Affiliated Brain Hospital of Guangzhou Medical University. The patients/participants provided their written informed consent to participate in this study.

## Author Contributions

Q-JK conceived and designed the analyses, performed the analyses, and wrote the manuscript. S-MZ contributed to the manuscript writing. YL contributed to the scientific editing and language editing of the manuscript. H-WW assisted in MRI data collection. T-YB and S-LS assisted in study conception and design. In addition, S-LS contributed to data collection. Y-JZ contributed to funding, conception, and design. All authors listed have made a substantial, direct, or intellectual contribution to the work, and approved it for publication.

## Conflict of Interest

The authors declare that the research was conducted in the absence of any commercial or financial relationships that could be construed as a potential conflict of interest.

## Publisher’s Note

All claims expressed in this article are solely those of the authors and do not necessarily represent those of their affiliated organizations, or those of the publisher, the editors and the reviewers. Any product that may be evaluated in this article, or claim that may be made by its manufacturer, is not guaranteed or endorsed by the publisher.
